# Partitioning the contributions of alternative malaria vector species

**DOI:** 10.1186/s12936-016-1107-y

**Published:** 2016-02-04

**Authors:** Anne Deredec, Samantha M. O’Loughlin, Tin-Yu J. Hui, Austin Burt

**Affiliations:** Department of Life Sciences, Imperial College, Silwood Park, Ascot, London, SL5 7 PY UK; UMR BIOGER, INRA, AgroParisTech, Université Paris-Saclay, 78850 Thiverval-Grignon, France

**Keywords:** Malaria transmission, Ross-McDonald, Vector control

## Abstract

**Background:**

In many locations malaria is transmitted by more than one vector species. Some vector control interventions, in particular those using genetic approaches, are likely to be targeted against a single species or species complex, at least initially, and it would therefore be useful to be able to predict the epidemiological impact of controlling a single species when multiple vector species are present.

**Methods:**

To address this issue, the classical Ross-McDonald model of malaria epidemiology is expanded to account for multiple vector species, giving expressions for the equilibrium prevalence, sporozoite rates and reproductive number. These allow one to predict when control of just one vector species will lead to elimination of the disease. Application of the model is illustrated using published data from a particularly extensive entomological and epidemiological survey before the rollout of bed nets in eastern Kenya, where *Anopheles gambiaes.l.* and *An. funestus* were vectors.

**Results:**

Meta-analysis indicates that sporozoite rates were 38 % higher in *An. gambiaes.l.* than in *An. funestus*, and, according to the model, this difference could be due to *An. gambiae s.l.* having a higher frequency of feeding on humans, a higher human-to-mosquito transmission rate, a lower adult mortality rate, and/or a shorter incubation period. Further calculations suggest that *An. gambiaes.l.* would have been sufficient to maintain transmission by itself throughout the region, whereas *An. funestus* would not have been able to support transmission by itself in Malindi District.

**Conclusions:**

Partitioning the contributions of different vector species may allow us to predict whether malaria will persist after targeted vector control.

## Background

Many indirectly-transmitted or vector-borne diseases are transmitted by more than one vector species, even within a single locale [1]. This is true of malaria, which in many places is transmitted by several species of anopheline mosquito [2–4]. Much of malaria control is directed against the mosquito vectors, as this has proven success in reducing malaria transmission and the burden of disease [5]. However, it is often the case that the intervention is more successful against some mosquito species than others. For example, bed nets and indoor residual spraying are more effective against species that bite and rest indoors than those that bite and rest outdoors, and as a consequence of their widespread adoption the relative importance of different vector species has shifted over time [6–11]. Some types of intervention currently under development, such as genetic approaches to vector control, are particularly likely to target just a single species or species complex, at least in the first instance [12, 13]. In thinking about potential species-specific interventions, it would be useful to be able to predict the epidemiological impact of controlling a single species when multiple vector species are present. How much will malaria transmission and prevalence be reduced by controlling a single species? When would it be possible to eliminate the disease if only a single species is targeted?

There has been a rich history of modelling the epidemiology of malaria, but the vast majority of it only considers a single vector species [14]. In this paper the classic Ross-McDonald model of malaria epidemiology [15, 16], modified to allow for super-infection and heterogeneity in propensity to be bitten [17, 18], is extended to allow for multiple vector species, leading to expressions for the equilibrium prevalence in humans, sporozoite rate in mosquitoes, and reproductive number. Application of the model is then illustrated using a particularly extensive survey of malaria and vectors in eastern Kenya [19].

## Methods

### Parasite prevalence in humans

Suppose there are *n* vector species, and for each species *i* let the number of adult female mosquitoes per human be *A*_*i*_, the average rate at which a female bites a person be *a*_*i*_, and the proportion of females that are infectious be *Y*_*i*_. Population sizes of the different vector species are assumed to be independently regulated such that changes in the density of one do not affect the others. The average rate at which a person is bitten by an infectious mosquito (i.e., the entomological inoculation rate) of species *i* is then *ɛ*_*i*_ = *a*_*i*_*A*_*i*_*Y*_*i*_. Each such bite is assumed to have a constant probability *b*_*i*_ of transmitting the infection to the person, which may differ among mosquito species. The average rate at which people acquire a new infection (i.e., the force of infection) is then the sum of the successfully infectious bites across vector species: $f = \sum\nolimits_{i = 1}^{n} {b_{i} \varepsilon_{i} }$. Suppose further that people differ in their propensity to be bitten, with person *j* having a relative propensity $s_{i\left( j \right)}$ by a mosquito of species *i*, such that the biting rate of infectious females of species *i* on this individual is *ɛ*_*i*_*s*_*i*(*j*)_. A person’s relative propensity to be bitten by mosquito species *i* is assumed to be gamma-distributed with mean 1 and variance *α*_*i*_. Individuals of propensity class *s* = {*s*_1_,···*s*_*n*_} then acquire new infections at a rate $f_{s} = \sum\nolimits_{i = 1}^{n} {s_{i} b_{i} \varepsilon_{i} }$. If multiple infections in an individual are cleared independently at a rate *r*, then the number of infections in an individual at any particular time is a Poisson variable with mean *f*_*s*_/*r*, and the overall clearance rate (i.e., rate at which an infected person becomes uninfected) is *f*_*s*_/(Exp[*f*_*s*_/*r*] − 1) (see [17] for the equivalent single species model). If *X*_*s*_ is the parasite prevalence in people with propensity *s* to be bitten, the change in *X*_*s*_ over time will then be:

$$\frac{{dX_{s} }}{dt} = \left( {1 - X_{s} } \right)f_{s} - \frac{{X_{s} f_{s} }}{{{\text{Exp}}\left[ {f_{s} /r} \right] - 1}}$$

At equilibrium, this expression equals 0, and

$$X_{s}^{*} = 1 - {\text{Exp}}\left[ { - f_{s} /r} \right] = 1 - {\text{Exp}}\left[ { - \mathop \sum \limits_{i = 1}^{n} s_{i} b_{i} \varepsilon_{i} /r} \right]$$

The equilibrium average prevalence in the population as a whole is then:

$$\bar{X}^{*} = \mathop \int \limits_{{{\mathbb{R}}_{ + }^{n} }}^{ } G\left( s \right)X_{s}^{*} ds = \mathop \int \limits_{0}^{\infty } \cdots \mathop \int \limits_{0}^{\infty } G\left( {s_{1} , \cdots ,s_{n} } \right)\left( {1 - e^{{ - \frac{{\mathop \sum \nolimits_{i = 1}^{n} s_{i} b_{i} \varepsilon_{i} }}{r}}} } \right)ds_{1} \cdots ds_{n}$$

where *G*(*s*) is the probability density function of the random vector *s* = {*s*_1_,···*s*_*n*_}. Evaluating this expression requires information or assumptions about how the propensity to be bitten by different vector species are correlated. Although there are several ways of constructing different families of multivariate gamma-distributions (i.e. distributions for which every marginal density is gamma-distributed), there is no general formulation for a multivariate gamma-distribution. Therefore, this paper focusses on two simple opposing cases: (I) an individual’s propensity to be bitten is equal for all vector species ($s_{i} \left( j \right) = s\left( j \right)$ for all $i$), with variance *α*; or (II) the propensities to be bitten by different species are independent of each other. In these two cases the equilibrium average prevalence is

1-I $$\bar{X}^{*} = 1 - \left( {\frac{r}{{r + \alpha \mathop \sum \nolimits_{i = 1}^{n} b_{i} \varepsilon_{i} }}} \right)^{1/\alpha }$$

1-II $$\bar{X}^{*} = 1 - \mathop \prod \limits_{i = 1}^{n} \left( {\frac{r}{{r + \alpha_{i} b_{i} \varepsilon_{i} }}} \right)^{{1/\alpha_{i} }}$$

respectively. Thus in both cases prevalence is an increasing function of *b*_*i*_ and *ɛ*_*i*_, and a decreasing function of *r* and *α*_*i*_.

### Sporozoite rate in mosquitoes

Suppose adult female mosquitoes of species *i*, in addition to having a constant probability of biting someone, *a*_*i*_, also have a constant probability of dying, *μ*_*i*_, both of which are independent of age and infection status. Let the probability a female mosquito becomes infected from biting an infected person be *c*_*i*_ (assumed to be independent of the number of infections the person carries). If it then takes *T*_*i*_ days for sporozoites to develop and the female to become infectious (the incubation period), the probability of her surviving this period is $\theta_{i} = e^{{ - \mu_{i} T_{i} }}$. The change in sporozoite rate in species *i* over time is then:

$$\frac{{dY_{i} }}{dt} = a_{i} c_{i} X_{{m_{i} }} \left( {\theta_{i} - Y_{i} } \right) - \mu_{i} Y_{i}$$

where $$X_{{m_{i} }} = \mathop \smallint \limits_{{{\mathbb{R}}_{ + }^{n} }}^{ } s_{i} G\left( s \right)X_{s}^{*} ds = \mathop \smallint \limits_{0}^{\infty } \cdots \mathop \smallint \limits_{0}^{\infty } s_{i} G\left( {s_{1} , \cdots ,s_{n} } \right)\left( {1 - e^{{ - \frac{{\mathop \sum \nolimits_{j = 1}^{n} s_{j} b_{j} \varepsilon_{j} }}{r}}} } \right)ds_{1} \cdots ds_{n}$$

is the parasite prevalence as seen from the mosquito’s point of view, or the probability of a mosquito biting an infected person (i.e., the weighted average prevalence, where the weights are the propensities to be bitten—see [20]). In the two specific cases studied, this index is:

2-I $$X_{{m_{i} }} = X_{m} = 1 - \left( {1 - \bar{X}^{*} } \right)^{1 + \alpha }$$

2-II $$X_{{m_{i} }} = 1 - \left( {1 - \bar{X}^{*} } \right) \cdot \frac{r}{{r + \alpha_{i} b_{i} \varepsilon_{i} }}$$

In the latter case, differences in the vector’s biting rate and/or in the entomological inoculation rate make the prevalence, as seen from a mosquito point of view, diverge among mosquito species even when all *α*_*i*_ are identical.

Setting *dY*_*i*_/*dt* equal to 0, the equilibrium sporozoite rate is:

3 $$Y_{i}^{*} = \frac{{a_{i} c_{i} X_{{m_{i} }} \theta_{i} }}{{a_{i} c_{i} X_{{m_{i} }} + \mu_{i} }}$$

Thus sporozoite rate is an increasing function of *a*_*i*_, *c*_*i*_ and $X_{{m_{i} }}$ and a decreasing function of *μ*_*i*_ and *T*_*i*_.

### Basic reproductive number (*R*_0_)

The basic reproductive number (*R*_0_) for a disease is the expected number of secondary human infections derived from a single primary infection in an otherwise uninfected population. This must be greater than 1 for the disease to persist indefinitely. If there are multiple vector species, it can be seen intuitively that the total number of secondary infections will be equal to the sum of the number transmitted by each individual vector species. That is,

$$R_{0} = \mathop \sum \limits_{i = 1}^{n} R_{0,i}$$

where *R*_0,*i*_ represents the contribution of the *i*th species to the total *R*_0_ (see also [21]).

Two different expressions can be given for the individual *R*_0,*i*_ values. First, in terms of the fundamental underlying parameters [20],

4 $$R_{0,i} = \frac{{a_{i}^{2} b_{i} c_{i} \theta_{i} A_{i} }}{{r\mu_{i} }}\left( {1 + \alpha_{i} } \right)$$

Note that if all the parameters *a*_*i*_*, b*_*i*_*, c*_*i*_*, T*_*i*_*, μ*_*i*_ and *α*_*i*_are equal between species, then a species’ proportionate contribution to *R*_0_ is equal to its proportionate contribution to the total number of mosquitoes, $A = \sum\nolimits_{i = 1}^{n} {A_{i} }.$ Alternatively, if lab data suggested, for example, that *b*_*i*_ for one species was half that for another, then, all else being equal, the ratio of their *R*_0_′s will be half the ratio of their abundances.

An alternative expression can be derived using the entomological inoculation rate, which, as noted above, is *ɛ*_*i*_ = *a*_*i*_*A*_*i*_*Y*_*i*_. Using Eq. (3) to substitute for *Y*_*i*_ gives:

$$\varepsilon_{i} = \frac{{a_{i}^{2} c_{i} \theta_{i} A_{i} X_{{m_{i} }} }}{{a_{i} c_{i} X_{{m_{i} }} + \mu_{i} }}$$

which can be rearranged to give:

5 $$\frac{{\varepsilon_{i} \left( {a_{i} c_{i} X_{{m_{i} }} + \mu_{i} } \right)}}{{X_{{m_{i} }} }} = a_{i}^{2} c_{i} \theta_{i} A_{i}$$

The terms in Eq. (4) corresponding to the right hand side of Eq. (5) can therefore be replaced by those on the left of (5), giving an expression for *R*_0,*i*_ in terms of *ɛ*_*i*_:

$$R_{0,i} = \frac{{\varepsilon_{i} \left( {1 + \frac{{a_{i} }}{{\mu_{i} }}c_{i} X_{{m_{i} }} } \right)b_{i} \left( {1 + \alpha_{i} } \right)}}{{rX_{{m_{i} }} }}$$

This expression assumes that the population is at equilibrium, and the underlying process is well described by the classical model. In the two cases studied, the species-specific basic reproductive number is thus:

6-I $$R_{0,i} = \varepsilon_{i} \frac{{b_{i} }}{r}\left( {\frac{{a_{i} c_{i} }}{{\mu_{i} }} + \frac{1}{{X_{m} }}} \right)\left( {1 + \alpha } \right)$$

6-II $$R_{0,i} = \varepsilon_{i} \frac{{b_{i} }}{r}\left( {\frac{{a_{i} c_{i} }}{{\mu_{i} }} + \frac{1}{{X_{{m_{i} }} }}} \right)\left( {1 + \alpha_{i} } \right) = \varepsilon_{i} \frac{{b_{i} }}{r}\left( {\frac{{a_{i} c_{i} }}{{\mu_{i} }} + \frac{{1 + \alpha_{i} \frac{{b_{i} \varepsilon_{i} }}{r}}}{{\bar{X}^{*} + \alpha_{i} \frac{{b_{i} \varepsilon_{i} }}{r}}}} \right)\left( {1 + \alpha_{i} } \right)$$

If the propensity to be bitten is equal for all species (case I), and if *a*, *b*, *c*, and *µ* are the same among vector species, then a species’ proportionate contribution to total *R*_0_ is equal to its proportionate contribution to total entomological inoculation rate. In this case eliminating a species that contributes a proportion *p* of the total number of infectious bites will reduce *R*_0_ to *R*_0_′ = *R*_0_(1−*p*). If that value is below 1, then the disease should be eliminated.

Further insight can be obtained by calculating for a particular vector species the ratio of entomological inoculation rate to reproductive number:

7-I $$\frac{{\varepsilon_{i} }}{{R_{0,i} }} = \frac{{rX_{m} }}{{\left( {1 + \frac{{a_{i} }}{{\mu_{i} }}c_{i} X_{m} } \right)b_{i} \left( {1 + \alpha } \right)}}$$

[22], where *X*_*m*_ is as defined in Eqs. (2-I, 2-II). This ratio is increased by increases in *X*, *r*, and *µ*, and decreased by increases in *a*, *c*, *b*, and *α*. These parameters will vary from population to population according to the local ecology and malaria control interventions, but for illustrative purposes if ‘exemplar’ values of *X* = 0.4, *r* = 0.01, *a* = 0.3, *b* = 0.5, *c* = 0.05, *µ* = 0.1, *α* = 4, (e.g., [5, 19, 23–26]) are used, and the result multiplied by 365 to convert to annual entomological inoculation rate, then a value of 1.2 is obtained. That is, in an area with multiple vector species and these parameter values, if one of the vectors has an annual entomological inoculation rate less than 1.2, then *R*_0,*i*_ for that species will be less than 1 and it would not be able to maintain transmission by itself.

This simple relationship between *R*_0,*i*_ and *ɛ*_*i*_ does not hold in case II, even if the distributions of the propensity to be bitten by the different vector species have identical variance. However it is still possible using Eq. (6-II) to derive the condition under which a species would be able to maintain transmission by itself. For the set of parameters value given above, the entomological inoculation rate should exceed 0.78 for malaria to persist if all other mosquito species are eliminated.

## Results

To illustrate the application of the multi-species model, a reanalysis was performed of the data presented in [19], a particularly extensive entomological and epidemiological survey of malaria in 30 villages in three Districts along the Indian Ocean coast of Kenya. In brief, malaria prevalence was estimated by a cross-sectional survey carried out at 30 primary schools, one per site, in May 1998. Blood smears were prepared from approximately 100 school children (aged 6–12 years) at each school. Mosquitoes were collected by pyrethrum spray catches (PSC) from inside 10 houses less than 2 km from each school. With few exceptions, the same houses were sampled once every two months from June 1997 to May 1998; collections occurred in the afternoons (noon to 3:00 p.m.). All mosquitoes were identified based on morphological characters, and mosquitoes in the *Anopheles gambiae s.l.* species complex were present at all 30 sites, while *An. funestus* was recovered from all but three sites. PCR identifications on a subsample of mosquitoes indicated that *An. gambiae s.s.* was the predominant member of the *An. gambiae* species complex in all villages except one, where *An. arabiensis* predominated. *An. arabiensis* was present in most villages, and *An. merus* in less than half. The heads and thoraces of all collected anophelines were tested for *Plasmodium falciparum* sporozoites using an enzyme-linked immunosorbent assay (ELISA). The entomological inoculation rate (*ɛ*_*i*_) was calculated by multiplying the proportion of sporozoite-positive mosquitoes (*Y*_*i*_) by the human biting rate, which in turn was calculated as the number of blood-fed and half-gravid mosquitoes collected by PSC divided by the number of persons sleeping in the house the night preceding the collections. Further details are given in [19].

### Analysis

For simplicity only the model of equal propensities to be bitten (case I) will be considered here. Also, because sporozoite rates were reported for the *An. gambiaes.l.* species complex as a whole rather than for the constituent species, the analysis is restricted to a comparison of *An. gambiaes.l.* and *An. funestus*. As these are the only two vectors, the total *R*_0_ will be the sum of the *R*_0_′s through each of them. If the reported values for *ɛ*_*i*_ and *X* are used, and otherwise the ‘exemplar’ parameter values above, then the expected total *R*_0_ can be estimated from Eq. (6-I). According to this calculation, the average *R*_0_ across villages was 14, 15, and 25 in Malindi, Kilifi, and Kwale Districts, respectively (n = 10 villages in each). In terms of partitioning the total *R*_0_ between *An. gambiae s.l.* and *An. funestus*, all else being equal the ratio of *R*_0_′s will be equal to the ratio of entomological inoculation rates (Eq. 6-I). The ratio of average entomological inoculation rates across the villages was $\bar{\varepsilon }_{g} /\bar{\varepsilon }_{f}$ = 48, 4.3, and 1.8 in the three districts (Table 1).

**Table 1:** Summary statistics from [19]

District	Prevalence (*X*)	Entomological inoculation rate		Reproductive number	
		*ɛ*_*g*_ (da^−1^)	*ɛ*_*f*_ (da^−1^)	*R*_0*g*_	*R*_0*f*_
Malindi	60.4	0.048	0.001	13.9	0.3
Kilifi	62.2	0.043	0.010	12.5	2.9
Kwale	64.3	0.056	0.032	16.2	9.3

Summary statistics for malaria prevalence and entomological inoculation rate from [19] plus estimated reproductive numbers calculated from Eq. (6-I), assuming *r* = 0.01, *a* = 0.3, *b* = 0.5, *c* = 0.05, *µ* = 0.1, and *a* = 4

**Fig. 1:**
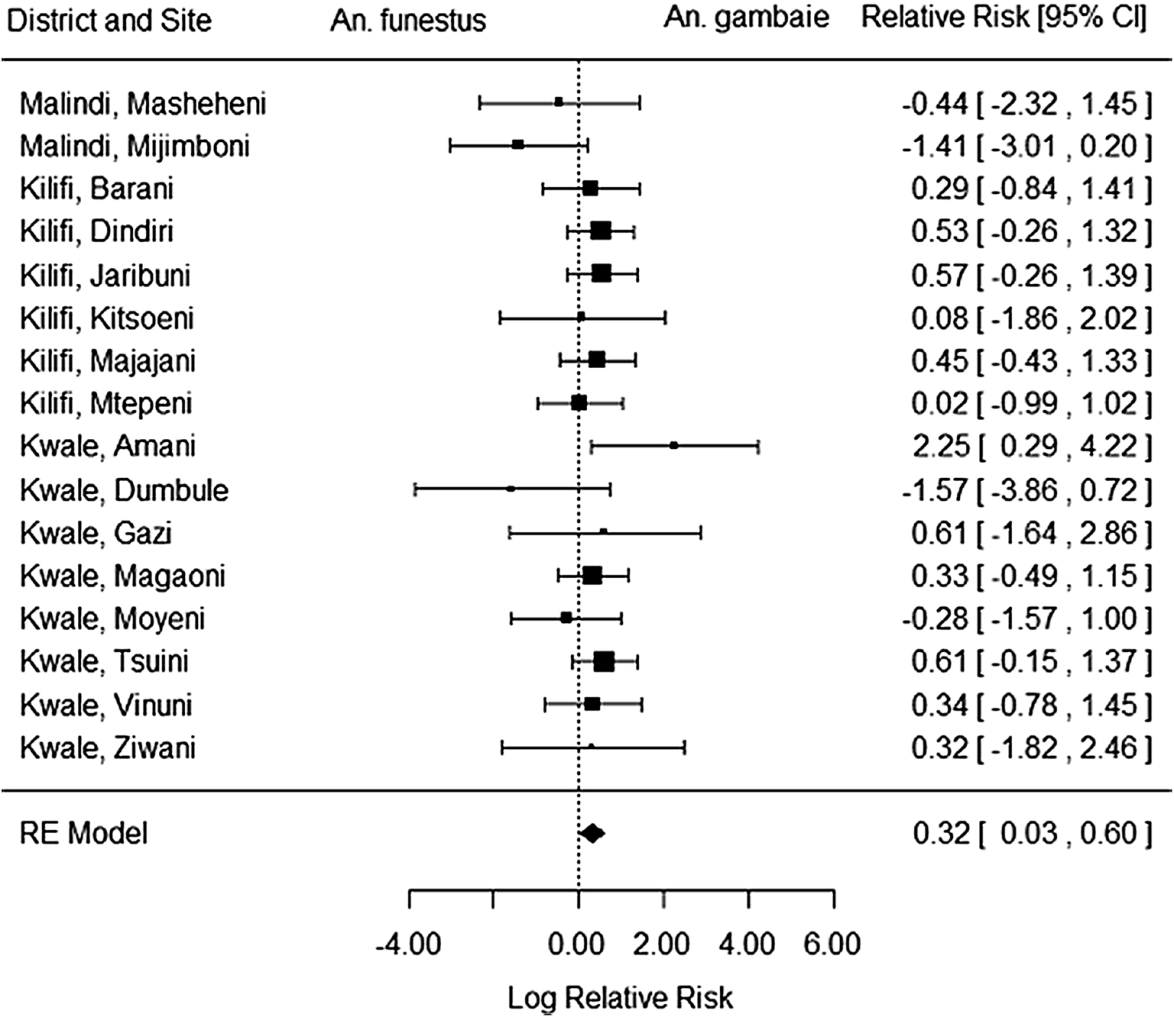
Forest plot of relative risk of *Anopheles gambiae* *s.l.* and *Anopheles funestus* being sporozoite positive across 16 villages in Eastern Kenya. Analysis performed using metafor [38]. Area of *black squares* represents the study’s weight in the meta-analysis

To investigate whether indeed ‘all else is equal’ between the two vector taxa, the simplest analysis is to test for differences in sporozoite rate. To do so while allowing for the variable sample sizes, the techniques of meta-analysis are used [27]. Fourteen villages were excluded from the analysis on account of no sporozoite-positive mosquitoes being found for one or both of the species (typically due to small sample sizes), and a random-effects model was used to analyse the log of relative risks from the remaining 16 villages. The analysis shows that the sporozoite rate of *An. gambiae* was e^0.32^ = 38 % higher than that of *An. funestus* (95 % CI 3–82 %), with no evidence of significant heterogeneity among villages around this estimate (Q = 14, df = 15, p = 0.52; Fig. 1).

**Fig. 2:**
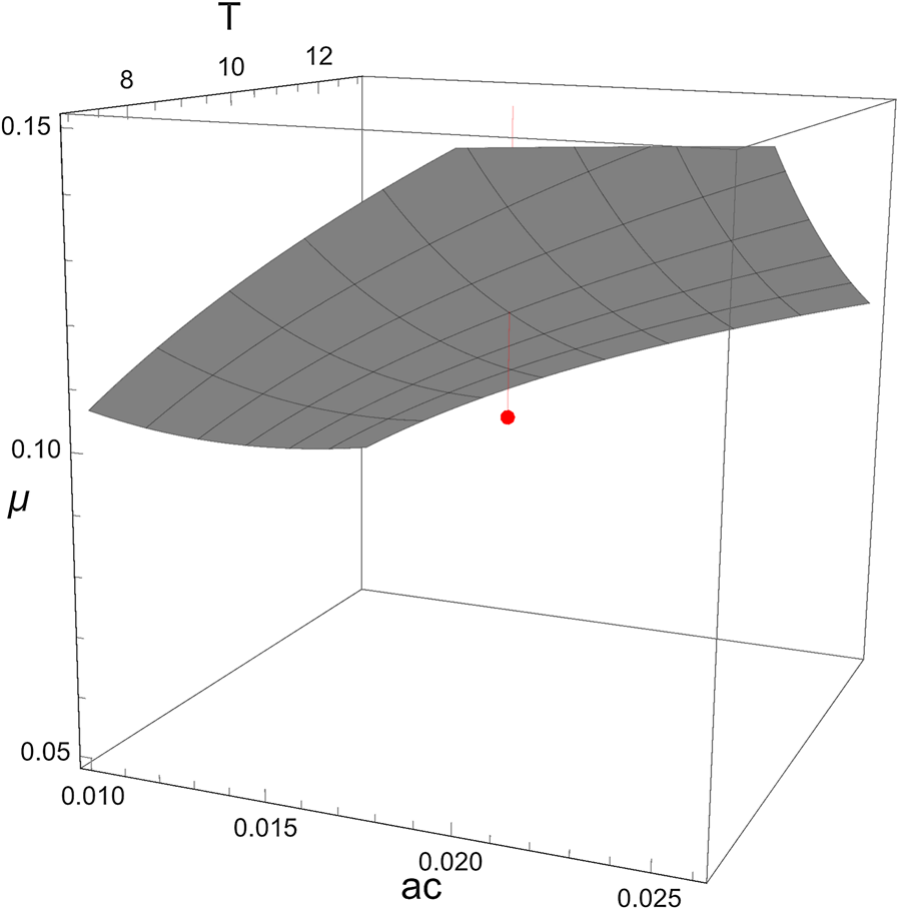
Three dimensional contour plot showing combinations of *ac*_*f*_, *µ*_*f*_, and *T*_*f*_ for *An. funestus* that would give an expected log sporozoite rate 0.32 less than that for *An. gambiae* s.l., assuming the latter has values indicated by the *red point* (*ac*_*g*_ = 0.018da^−1^, *µ*_*g*_ = 0.1da^−1^ and *T*_*g*_ of 10da, which together give an expected sporozoite rate of 5.6 %, equal to the observed average rate for this species)

This difference in sporozoite rate between species suggests that at least one of the underlying parameters of Eq. (3) differs between the species: *An. gambiae s.l.* has a higher frequency of feeding on humans (*a*), a higher human-to-mosquito transmission rate (*c*), a lower adult mortality rate (*µ*), and/or a shorter incubation period in these populations (*T*). Alternatively, the structure of the model could be wrong, and, for example, *An. gambiaes.l.* feeds on people that have a higher prevalence than does *An. funestus,* or predominates at times of the year when prevalence is higher, but these alternatives will not be pursued here. To give an idea of what sorts of differences could account for a 38 % difference in sporozoite rate, Eq. (3) is used, noting that the average prevalence was $\bar{X}^{*}$ = 62.3 % , giving *X*_*m*_ = 0.99 (Eq. (2-I), assuming *α* = 4). For example, if *An. gambiae s.l.* has values of *ac*_*g*_ = 0.018da^−1^, *µ*_*g*_ = 0.1da^−1^ and *T*_*g*_ of 10da, giving an expected sporozoite rate of 5.6 %, equal to the observed average rate for this species, then the necessary decrement for *An. funestus* could be gotten by any one of the following changes: *ac*_*f*_ = 0.012da^−1^, *µ*_*f*_ = 0.118da^−1^ or *T*_*f*_ = 13da (for combinations of parameter values giving the requisite difference, see Fig. 2). The average prevalence in the population as a whole is probably lower than that for school children [18], but virtually identical results are obtained if $\bar{X}^{*}$ = 40 % . Differences between the species in *c* or *T* could be investigated in the laboratory (for comparisons within the *An. gambiae* species complex, see [28–32], but for *a* or *µ* one would need to work in the field.

This difference in sporozoite rates suggests that bites by *An. gambiae s.l.* may contribute more to malaria prevalence than bites by *An. funestus*. In principle, this effect could be detected in an analysis of the relationship between malaria prevalence and human biting rate by the two species. However, in this particular dataset there is no significant relationship between prevalence and biting rate by either species, or total biting rate, presumably because the villages are past the point of saturation for malaria transmission.

What effect do the differences in *ac*, *µ* or *T* suggested by the sporozoite rate comparison have on the estimate of the ratio of *R*_0_′s? Using Eq. (6-I), if the above example values for *ac*_*g*_ and *ac*_*f*_ are used, then *R*_0*g*_/*R*_0*f*_ = 1.05*ɛ*_*g*_/*ɛ*_*f*_. If the above values for *µ*_*g*_ and *µ*_*f*_ are used, then *R*_0*g*_/*R*_0*f*_ = 1.02*ɛ*_*g*_/*ɛ*_*f*_. Finally, the incubation period *T* does not appear explicitly in Eq. (6-I) (its effect is wholly through *ɛ* and *X*_*m*_), and, therefore, if the difference in sporozoite rates is due solely to differences in *T*, then *R*_0*g*_/*R*_0*f*_ = *ɛ*_*g*_/*ɛ*_*f*_. In any of these cases the adjustment is small compared to other uncertainties in the estimation and analysis, and *ɛ*_*g*_/*ɛ*_*f*_ is a reasonable estimate of *R*_0*g*_/*R*_0*f*_.

Finally, the average estimated *R*_0_ through *An. gambiaes.l.* was greater than 12 in each of the three districts, indicating it should be able to maintain malaria transmission by itself (Table 1). *R*_0_ through *An. funestus* was smaller than through *An. gambiaes.l.*, but still greater than 1 in Kilifi and Kwale, but only 0.3 in Malindi, suggesting that in this district it would be incapable of supporting transmission by itself, and elimination of *An. gambiaes.l.* would be sufficient to eliminate the disease. It is worth noting that these data were collected before the large-scale deployment of bed nets in the area. Mosquito abundances and entomological inoculation rates are now very much lower [9] and, therefore, estimates of *R*_0_ would also be much lower.

## Conclusions

In many places malaria is transmitted by more than one vector species, and vector control interventions are likely to have different effects on the different species. To better understand the overall impact of an intervention in these circumstances, the classic Ross-McDonald model has been expanded to include multiple vector species. This model was then used to guide a re-analysis of a particularly extensive study in eastern Kenya. This re-analysis indicates that *An. gambiaes.l.* had a 38 % higher sporozoite rate than *An. funestus* in this area, and the model suggests this difference could be due to higher human biting rate or efficiency of parasite acquisition, or reduced death rate or incubation period for the parasite. Differences between the taxa in transmission efficiencies or incubation periods could be investigated in the laboratory, but for the other parameters one would need to work in the field.

The total reproductive number (*R*_0_) for malaria in a region will be the sum of the *R*_0_′s through the individual vector species. All else being equal, the relative contributions of the different vectors to total *R*_0_ will be equal to their relative contributions to the total entomological inoculation rate. Even in the Kenyan study where the differences in sporozoite rate indicated not all else was equal, this seems a good approximation. Further calculations with this pre-bed net dataset suggest that *An. gambiae s.l.* by itself would have been sufficient to maintain transmission throughout the region studied, but that in Malindi, *An. funestus* could not have maintained transmission by itself: elimination of *An. gambiae s.l.* would have led to elimination of the disease.

As the modelling is a straight-forward extension of the classic Ross-McDonald model, it shares the same advantages and disadvantages [33], and it would be interesting to address these questions in more fine-grained models [34, 35]. In terms of the propensities to be bitten, two simple and opposing cases have been considered, where they are either equal or uncorrelated across species. A more general approach would allow unequal but correlated values, and several ways of constructing such multivariate gamma distributions have been developed in the past [36, 37]. The modelling has also assumed that there are no significant ecological interactions between the vector species (other than transmitting the same parasite), and the model could be extended to allow for competitive release, predator switching, or other such interaction. The real test, of course, will come with the deployment of genetic or other species-specific vector interventions, accompanied by careful field observations.
